# Glycosylation End-product Formation in Retinal Membranes, an Energy Alteration Injury

**Published:** 2012

**Authors:** Viroj Wiwanitkit

**Affiliations:** Adjunct professor, Joseph Ayobabalola University, Nigeria, Visiting Professor, Hainan Medical University, China

**Dear Editor,**

Poorly controlled diabetes mellitus can lead to blindness due to severe proliferative diabetic retinopathy. The pathophysiology of retinal involvement is important in diabetic retinopathy, with stress-induced cellular ion change and oxidation generally proposed to be the underlying mechanism [1 – 2]. Based on the concept of nanomedicine, it has been proven that there is no alteration in flux crossing the retina membrane in diabetes mellitus, so this cannot be the cause of retinopathy [3]. The author hereby proposed a new theory that there must also be an underlying problem in the cell directly. Indeed, retina cells also contain the protein, and this could be the attack point for hyperglycaemic bombardment. Applying the concept of energy fluctuation due to a high blood glucose-induced glycosylation process [4], the pathology of the retina might be explained. Indeed, the discovery of glycosylation processes in retinal cells has just been reported [5], which confirms the opinion of the energy fluctuation-induced retinal problems in poorly controlled diabetic cases [4]. This hypothesis could be justified by further basic clinical investigations.

## DISCLOSURE

The authors report no conflicts of interest in this work.
